# Epigenome-wide methylation analysis of colorectal carcinoma, adenoma and normal tissue reveals novel biomarkers addressing unmet clinical needs

**DOI:** 10.1186/s13148-023-01516-7

**Published:** 2023-07-06

**Authors:** Katleen Janssens, Isabelle Neefs, Joe Ibrahim, Anne Schepers, Patrick Pauwels, Marc Peeters, Guy Van Camp, Ken Op de Beeck

**Affiliations:** 1 Centre of Medical Genetics, University of Antwerp and Antwerp University Hospital, Prins Boudewijnlaan 43, 2650 Edegem, Belgium; 2 Centre for Oncological Research Antwerp (CORE), University of Antwerp and Antwerp University Hospital, Universiteitsplein 1, 2610 Wilrijk, Belgium

**Keywords:** Colorectal cancer, Methylation, Biomarkers, Adenoma, Carcinoma

## Abstract

**Background:**

Biomarker discovery in colorectal cancer has mostly focused on methylation patterns in normal and colorectal tumor tissue, but adenomas remain understudied. Therefore, we performed the first epigenome-wide study to profile methylation of all three tissue types combined and to identify discriminatory biomarkers.

**Results:**

Public methylation array data (Illumina EPIC and 450K) were collected from a total of 1 892 colorectal samples. Pairwise differential methylation analyses between tissue types were performed for both array types to “double evidence” differentially methylated probes (DE DMPs). Subsequently, the identified DMPs were filtered on methylation level and used to build a binary logistic regression prediction model. Focusing on the clinically most interesting group (adenoma vs carcinoma), we identified 13 DE DMPs that could effectively discriminate between them (AUC = 0.996). We validated this model in an in-house experimental methylation dataset of 13 adenomas and 9 carcinomas. It reached a sensitivity and specificity of 96% and 95%, respectively, with an overall accuracy of 96%. Our findings raise the possibility that the 13 DE DMPs identified in this study can be used as molecular biomarkers in the clinic.

**Conclusions:**

Our analyses show that methylation biomarkers have the potential to discriminate between normal, precursor and carcinoma tissues of the colorectum. More importantly, we highlight the power of the methylome as a source of markers for discriminating between colorectal adenomas and carcinomas, which currently remains an unmet clinical need.

**Supplementary Information:**

The online version contains supplementary material available at 10.1186/s13148-023-01516-7.

## Introduction

Colorectal cancer (CRC) is one of the leading causes of cancer-related deaths worldwide. More than 1.9 million incidence cases and over 935 000 deaths were observed worldwide in 2020 [1]. In early stages (I and II), 5-year overall survival chances are at least 90%. Detection of CRC in an advanced stage (IV) reduces survival chances to only 15% [2, 3]. Unfortunately, 75% of all CRC tumors are discovered in advanced stages. Therefore, early detection of colorectal tumors must clearly improve.

CRC is a very heterogeneous disease that typically develops from pre-cancerous lesions, so-called adenomas. In 80% of cases, CRC develops through the adenoma-carcinoma sequence, a process that can take up to 10 years since adenomas tend to progress slowly, with increasing size and dysplasia over time [4, 5]. It has already been demonstrated that both genetic and epigenetic alterations are acquired in the tumor genome during carcinogenesis [5]. Epigenetic alterations have been studied over the past years and have revealed the relation between specific gene expression patterns apart from genetic mutations [5, 6].

One of the most studied epigenetic modifications is DNA methylation. In CRC, widespread hypomethylation blocks have been observed, as well as hypermethylation of specific CpG islands in gene-specific promotors [5, 7]. Despite many efforts, there is still a lot to discover at a molecular level for methylation in colorectal tissue. Particularly, methylation patterns in precancerous colorectal lesions, notably adenomas, are understudied. Online available datasets such as The Cancer Genome Atlas (TCGA) or Gene Expression Omnibus (GEO) mostly include methylation data of invasive tumor tissue. As methylation occurs in very early stages of carcinogenesis, DNA methylation biomarkers are the most compelling candidates for early detection of cancer [5]. Therefore, the DNA methylation data of adenomas are of extreme importance.

In previous research [7], it was already demonstrated that normal tissue and colorectal cancer tissue can be discriminated based on differentially methylated CpG sites. The study was based on publicly available data, which lacks the information on methylation of precancerous lesions as described earlier. Other researchers [5] have investigated differential methylation in normal and low-grade versus high-grade adenomas. Although this study shows very promising results for early biomarker candidates, it lacks a comparison with colorectal cancer tissue. Up until this moment, there is no possibility to discriminate colorectal adenomas from adenocarcinomas with molecular biomarkers in the clinic. However, such biomarkers would be an interesting and important tool for earlier described reasons.

To our knowledge, epigenome-wide analysis of normal, adenoma and colorectal tumor tissue has never been performed simultaneously. Therefore, the goals of this study were: to 1) explore and compare the epigenome of normal colorectal tissue, adenomas and colorectal tumor tissue in one experiment and 2) to identify molecular biomarkers that can discriminate especially between colorectal carcinoma and adenoma. Based on currently available data, we hypothesized that each of the three tissue types would have a different methylation pattern.

## Methods

### Study population, sample collection and pathologist review

A total of 55 samples were requested at the Biobank and the pathology department of the Antwerp University Hospital. Three different types of samples were used: 19 normal adjacent, 17 adenoma and 19 colorectal tumor tissue samples. This included 10 pairs of colorectal cancer and normal samples and 1 pair of adenoma-normal samples of the same patient. Tissue specimens were formalin-fixed paraffin-embedded (FFPE). For each specimen, 10 sections of 6 µm were made of which one slide was stained with hematoxylin and eosin for histologic review. A pathologist verified the tissue type and estimated the percentage of tumor cells. To limit the contamination by non-tumor cells, macrodissection was performed where possible. All samples had at least 50% tumor cells.

### DNA extraction and processing

DNA was isolated using the QIAamp FFPE Tissue kit (Qiagen, Hilden, DE) according to the manufacturer’s instructions. It is known that FFPE samples generally perform poorly on array-based applications due to the highly degenerated DNA. Therefore, the quality of the DNA was verified using the Infinium FFPE QC kit (Illumina Inc., San Diego, CA, USA) according to the manufacturer’s protocol. Only samples with good amplification for all replicates and a maximal ΔCq (difference in quantification cycles compared to the standard) below 5 were selected for use in the bisulfite conversion and restoration step. Bisulfite conversion was performed using the EZ DNA Methylation kit (Zymo Research, Freiburg im Breisgau, DE), according to the manufacturer’s instructions. The array-specific incubation program was used for all samples. After bisulfite conversion, DNA samples were restored using the Infinium HD FFPE Restoration kit (Illumina Inc.).

### In-house experimental methylation dataset

In total, 55 clinical samples were obtained and processed, the details of which are available in Additional file 1: Tables 1 and 2. The Illumina Human MethylEPIC® v1.0 BeadChip (Illumina Inc.) [8] was used to interrogate more than 850 000 CpG sites (probes) genome-wide at single-nucleotide resolution. Raw intensity array data were processed using the minfi (v 1.42.0) R package [9]. Methylation levels were reported as β-values ranging from 0 for unmethylated probes to 1 for fully methylated probes. For quality control, the ratio of log2 median intensities (methylated and unmethylated) along with β-value densities was calculated. β-values were then further preprocessed using ChAMP (v 2.21.1) [10] where probes with a detection *p*-value > 0.01 in more than 50% of the samples were removed. Control probes, X-/Y chromosome probes, multihit probes, and probes with known single nucleotide polymorphisms (SNPs) were filtered out of the analyses. BMIQ normalization was used to reduce the technical variation of Type-I and Type-II Illumina probes [11]. Out of 55 samples, 28 samples failed quality check and were removed from downstream analyses. The final analyses included 27 samples with 740 330 autosomal probes each (Additional file 1: Table 2).

**Table 1:** Summary of DMPs, DMRs and DMBs in all three analyses

	Comparison	Adenoma versus normal	Carcinoma versus normal	Adenoma versus carcinoma
DMB	EPIC	703	582	1552
DMR	EPIC	3510	6756	5067
DMP	450K	344,165	304,548	170,300
	EPIC	620,643	693,813	558,897
	Common DMP (450K and EPIC)	257,141	258,853	124,082
	DE DMP with │Δβ│ ≥ 0.3 in EPIC AND 450K	62	56	**13**

Bol value indicate *p* value ≤ 0.01

### Public methylation datasets

Array data from both Illumina Infinium HumanMethylation450 (more than 450 000 CpG sites) and Human MethylEPIC® BeadChips were downloaded from several public data repositories including GEO, TCGA and the Array Express databases. A total of 1 116 450K and 786 EPIC samples were acquired, the accession numbers and full details of which can be found in Additional file 1: Table 3. To ensure consistent data processing, we opted to use signal intensity or raw idat files. The datasets were then processed using the same steps described above for the in-house experimental methylation data. Out of the total 1 879 samples, 14 failed quality check and were removed from downstream analyses.

### Ethical approval

The study was conducted under Good Clinical Practice guidelines and the Declaration of Helsinki. Samples used in this study were previously collected in the Biobank of the Antwerp University Hospital and retrospectively used in this study. Patients give consent for the use of their bodily material in research when consenting to an invasive procedure (according to article 20 of the Belgian Law on the procurement and use of human corporal material intended for human application or scientific research of 19 December 2008). Approval for the study protocol (and any modifications thereof) was obtained from the ethical committee of the Antwerp University Hospital (Ref. N°20/02/010). Other data used in this study are publicly available. As such, neither patient consent nor institutional review board approval was required.

### Definitions of genomic regions and differential methylation

Genomic region annotations were based on Illumina 450K and EPIC array manifest files and were divided into two main groups. The first consists of genomic locations concerning genes. These included: 1st exon; 3′ UTR (3′ untranslated region), 5′ UTR (5′ untranslated region), Body (gene body), IGR (intergenic regions), TSS1500 (200 to 1500 nucleotides, upstream of the transcription start site, TSS), TSS200 (up to 200 nucleotides upstream of TSS), and ExonBnd (exon boundaries). The second describes annotations of probe location relative to CpG islands. These included: Islands, North shelf (2–4 kb upstream of CpG island), North shore (0–2 kb upstream of CpG island), Open Sea (non-CpG island-related sites), South shelf (2–4 kb downstream of CpG island), and South shore (0–2 kb downstream of CpG island).

Genome-wide DNA methylation was investigated in the context of differentially methylated probes (DMPs), regions (DMRs) and blocks (DMBs). DMPs were defined as CpG sites with statistically significant differences in methylation levels between groups. In contrast, DMRs and DMBs are larger genomic regions—between ~ 10 bp—10 kb and 10 kb—1 Mb, respectively—exhibiting a quantifiable difference in methylation between groups and containing hundreds of CpG sites.

### Differential methylation analyses

**Fig. 1:**
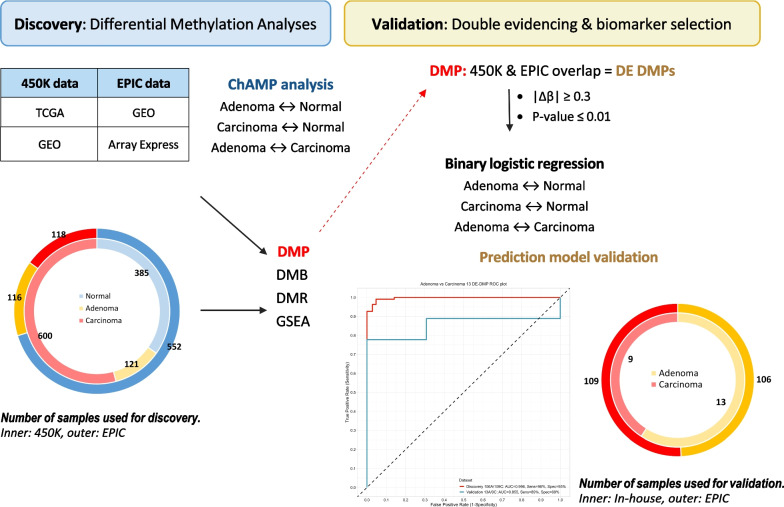
Study overview. *DMP* differentially methylated probe, *DMB* differentially methylated block, *DMR* differentially methylated region, *GSEA* gene set enrichment analysis, *DE* double evidenced

Differential methylation analysis was carried out via ChAMP (v 2.21.1), which uses parametric linear mixed models to test differences in methylation between groups [10]. A two-level, three-way differential methylation analysis was performed in the public EPIC datasets; adenoma versus normal tissue, carcinoma versus normal tissue, and adenoma versus carcinoma (Fig. 1). DMP *p*-values were adjusted for multiple testing using the Benjamini–Hochberg correction. DMRs and DMBs were identified using an implemented extension of the Bumphunter algorithm in ChAMP, with minimum sizes of 50 and 500 bp, respectively. Gene set enrichment analysis (GSEA) was done using the ChAMP and methylGSA R packages [12]. Differential methylation analysis was carried out on the public methylation datasets which constituted the discovery cohort (Fig. 1).

### Double evidencing and biomarker selection

DMPs that were first identified through the EPIC differential methylation analyses and that were later also found in the 450K analyses were termed double evidenced DMPs (DE DMPs). These DE DMPs represent important methylation markers that are identified through the analysis of two separate, large datasets. The criteria for assigning DE DMPs for use in the subsequent models were 1) a |Δβ|≥ 0.3 [13] and 2) a corrected *p*-value ≤ 0.01 in both array types. After merging DMP lists and screening for DE DMPs, binary logistic regression models were fitted to predict tissue type (adenoma/carcinoma/normal tissue) using the specific DE DMPs for each of the three analyses (Fig. 1). To test over-fitting, all models were tenfold cross-validated. Prediction metrics were assessed by plotting receiver operating characteristic (ROC) curves, and confusion matrices were also generated to calculate overall sensitivity, sensitivity and accuracy. The final model was then validated in the in-house experimental methylation datasets which constituted the validation cohort. Prediction metrics were also calculated for the validation model.

### Statistical analyses

The statistical software R (v 4.2.0) [14] was used for all analyses and visualizations. In all regression models, age was accounted for as a covariate, but was excluded from the final model if its effect on the outcome was not significant. Unless stated otherwise, all reported *p*-values are two-sided, and those ≤ 0.01 were considered statistically significant. All genomic annotations were based on the GRCh37/hg19 genome build.

## Results

### Genome-wide methylation profiling

**Fig. 2:**
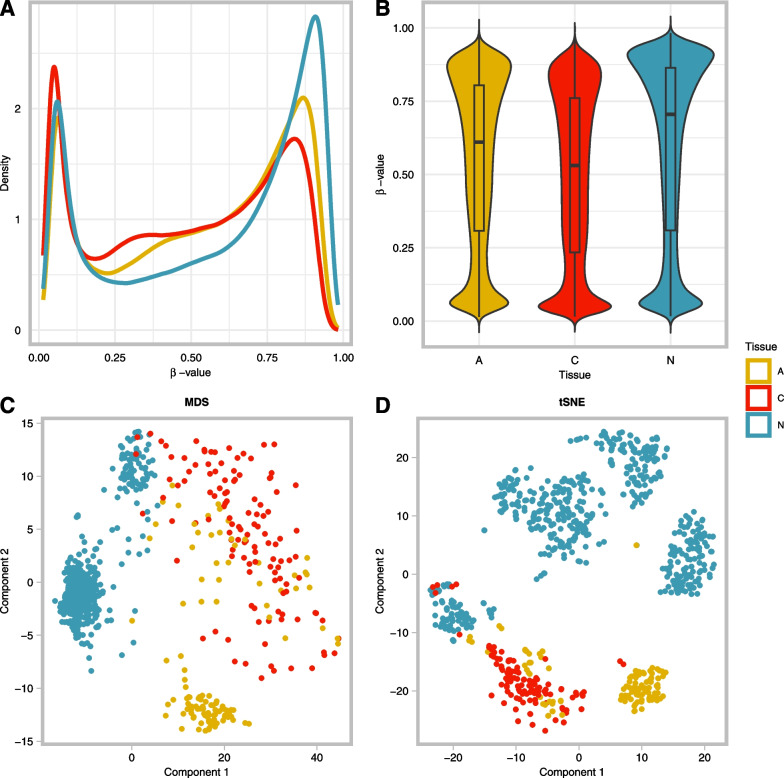
Landscape of DNA methylation of adenoma, carcinoma, and normal colorectal tissues in EPIC datasets. **A** Density plot showing the characteristic bimodal distribution of methylation β-values in all 3 tissues based on EPIC array data. **B** Violin plot of the mean methylation in each of the tissues, shows overall methylation decreases with increase in malignancy. **C** MDS plot highlighting the data structure and sample relationship among the tissue groups in EPIC array data. **D** tSNE plot showing a defined cluster for each of the different tissues, highlighting the ability to resolve samples based on their methylation patterns, despite overlap between adenomas and carcinomas. *MDS* multidimensional scaling, *tSNE* t-distributed stochastic neighbor embedding, *A* adenoma, *C* carcinoma, *N* normal tissue

To comprehensively explore the difference in methylation patterns between normal, adenoma and carcinoma tissue, DNA methylation was profiled pairwise between the three tissue types. This genome-wide differential methylation profiling was carried out on public EPIC array datasets. The results of these analyses are summarized in Table 1. Sizeable genome-wide DNA methylation differences were observed between the three tissue types (Fig. 2). β-values in all three tissues exhibited characteristic bimodal distributions (Fig. 2A), while on average normal tissues had the highest methylation levels followed by adenomas and lastly carcinomas (Fig. 2B). Based on the widespread differences in methylation, the three tissues clustered independently using both multidimensional scaling (MDS) and t-distributed stochastic neighbor embedding (tSNE) approaches (Fig. 2C, D). MDS is used for the visualization of outliers, while tSNE rather shows how samples group together. In our analyses, both methods agreed. The tSNE plot shows four distinct clusters for normal tissue (N). The tissues formed mostly discernable clusters where (pre)malignant lesions (i.e., adenomas (A) and carcinomas (C)) could be clearly resolved from N. However, A and C clustered more closely together (Fig. 2C, D).

### DMPs

**Fig. 3:**
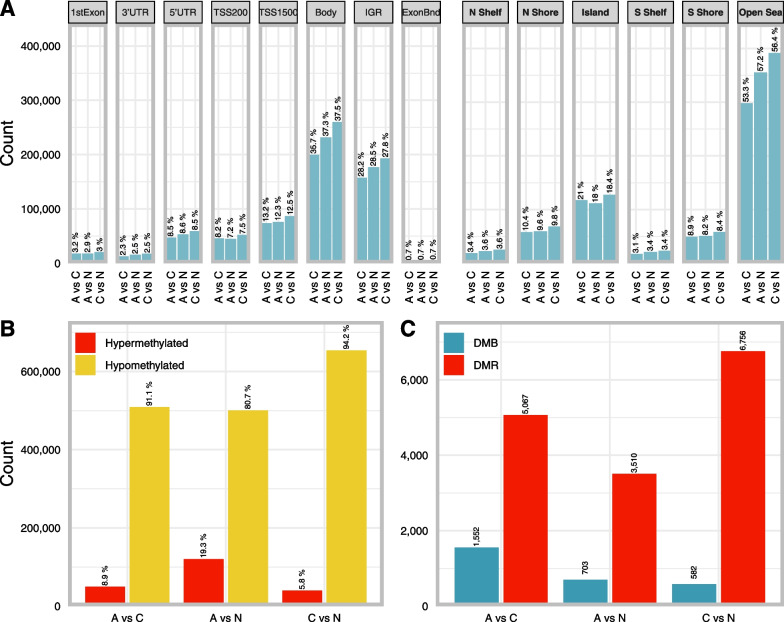
Differential methylation in adenoma, carcinoma, and normal colorectal tissues in both EPIC and 450K datasets. **A** Barplot of DMP counts per genomic region for each of the 3 pairwise comparisons for both methylation platforms. Percentages are fractions of the total DMPs for each comparison and platform. **B** Barplot of hyper- (β ≥ 0.7) and hypomethylated (β ≤ 0.3) DMPs for each of the 3 comparisons for both methylation platforms. Percentages are fractions of the total DMPs for each comparison and platform. **C** Barplot of DMB and DMR counts for all 3 comparisons for both methylation platforms. Annotations in regular font are with reference to genes, those in bold are with reference to CpG islands. *UTR* untranslated region, *IGR* intergenic region, *TSS* transcription start site, *N* north, i.e., upstream (5’) of CpG island, *S* south, i.e., downstream (3’) of CpG island, *ExonBnd* exon boundaries, *DMB* differentially methylated block, *DMR* differentially methylated region

When studying differences in DNA methylation at a single-base resolution, we identified 620 643 DMPs in A vs C. When C vs N was compared, 693 813 DMPs were observed while comparing A vs N resulted in 558 897 DMPs (see EPIC data in Table 1). The distribution and location of these DMPs in relation to genomic features and CpG islands are shown in Fig. 3A. In each comparison, most DMPs were in the gene body (36.84% on average), which is expected based on the distribution of probes on the EPIC array [15]. This was followed by the intergenomic regions (28.16% on average) and TSS1500 (12.66% on average). We also found DMPs located in the 5’UTR (8.53% on average), in the TSS200 (7.64% on average), the 1st exon (3.04% on average) and 3’UTR (2.44% on average). Lastly, the exon boundaries were studied, but they only represented 0.68% of DMPs (Fig. 3A). Concerning DMP location in relation to CpG islands, the largest proportion of DMPs mapped to open-sea regions (55.66% on average) followed by CpG islands (19.13% on average). North shores contained ± 9.92% of DMPs, while south shores contained on average 8.47% of DMPs. North and south shelves contained the lowest average proportion of DMPs at 3.53% and 3.28%, respectively (Fig. 3A). Definitions of DMP locations can be found in the materials and methods section.

The majority of DMPs were hypomethylated compared to hypermethylated (80.67% in A vs N, 94.21% in C vs N and 91.08% in A vs C, in Fig. 3B). When evaluating the tissue types, most DMPs were hypomethylated in tissue types with a higher degree of malignancy (given that the malignant potential increases from normal, to adenoma and eventually carcinoma) (Fig. 3B). To allow for a comparison between the three tissue types, the DMP counts are normalized by dividing them through the total number of analyzed CpG sites in each category.

### DMRs and DMBs

To study small regions with differential methylation that might be functionally involved in transcriptional regulation, DMRs between the three tissue types were studied. Most DMRs were identified when C vs N were compared, followed by the comparison of A vs C and the smallest number of DMRs were identified when A vs N were compared (6 756, 5 076 and 3 510 DMRs, respectively) (Fig. 3C). Since it has been reported that large hypomethylated blocks are a universal feature of cancer tissue, methylation data was analyzed to identify DMBs for the comparison of the three tissue types. We identified 1 552 DMBs when comparing A vs C, 703 DMBs when comparing A vs N and lastly 582 DMBs for C vs N (Fig. 3C). Definitions of DMRs and DMBs can be found in the materials and methods section “Definitions of genomic regions and differential methylation”.

### Double evidenced differential methylation (DE DMPs)

To double evidence the DMPs identified through the public MethylEPIC® dataset, analysis of additional Illumina 450K data of 385 normal, 121 adenoma and 600 carcinoma samples from public datasets was performed (1 106 450K samples mentioned in Fig. 1). The common DMPs that were detected in the datasets of both the EPIC and 450K methylation arrays and had an absolute delta beta value of > 0.3, were termed double evidenced DMPs (DE DMPs). Additional file 1: Fig. 1 represents an overview of the unique and common DMPs in the three different tissue groups. Sixty-two DE DMPs were identified when comparing adenoma and normal tissue, 56 DE DMPs for carcinoma and normal tissue and 13 DE DMPs for adenoma and carcinoma tissue (shaded row in Table 1). More information regarding the location of the DE DMPs within the genome can be found in Additional file 2: Table 4.

### Methylation as a biomarker for adenomas and carcinomas

**Fig. 4:**
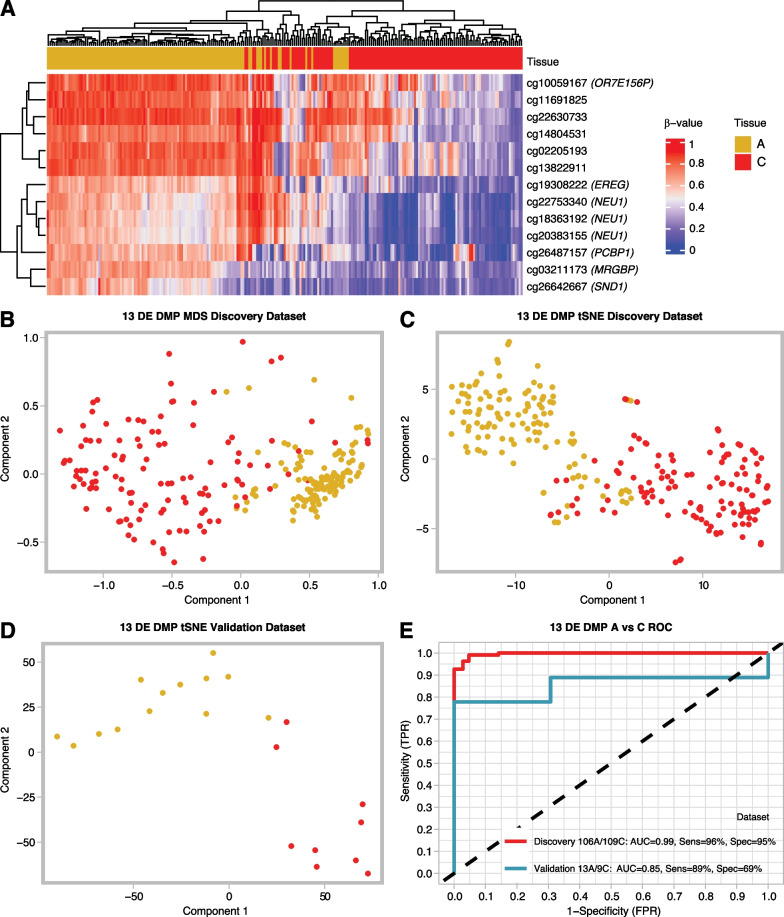
The selected 13 DE DMP markers were effective at classifying adenomas and carcinomas. **A** Heat map and hierarchical clustering analysis of the discovery EPIC dataset based on the 13 identified DE DMP markers shows a block like structure with almost half of the markers being hypermethylated in carcinoma and hypomethylated in adenomas and vice versa for the other half. **B** MDS clustering of the discovery dataset using the 13 markers shows 2 distinct clusters. **C** tSNE clustering of the discovery dataset using the 13 markers could also resolve the two tumor types. **D** tSNE clustering of the validation dataset using the 13 markers shows a clear separation between adenomas and carcinomas, only 2 carcinomas are falsely classified. **E** ROC curves for the final 13 DE DMP classifier model for both discovery and validation datasets from EPIC arrays. Sensitivity and specificity, for distinguishing between adenomas and carcinomas, at various cut-off values for the datasets are plotted. The model yielded an AUC of 0.99 and reached a sensitivity and specificity of 96.33% and 95.28%, respectively, while overall model accuracy was 95.81% in the discovery dataset. In the validation dataset it had an AUC of 0.85, and reached a sensitivity and specificity of 89.36% and 69.78%, respectively. The diagonal dotted line represents the line of no discrimination between the two tumor types. *DE DMP* double evidenced differentially methylated probes, *ROC* receiver operating characteristic, *MDS* multidimensional scaling, *tSNE* t-distributed stochastic neighbor embedding, *TPR* true positive rate, *FPR* false positive rate, *A* adenoma, *C* carcinoma

**Fig. 5:**
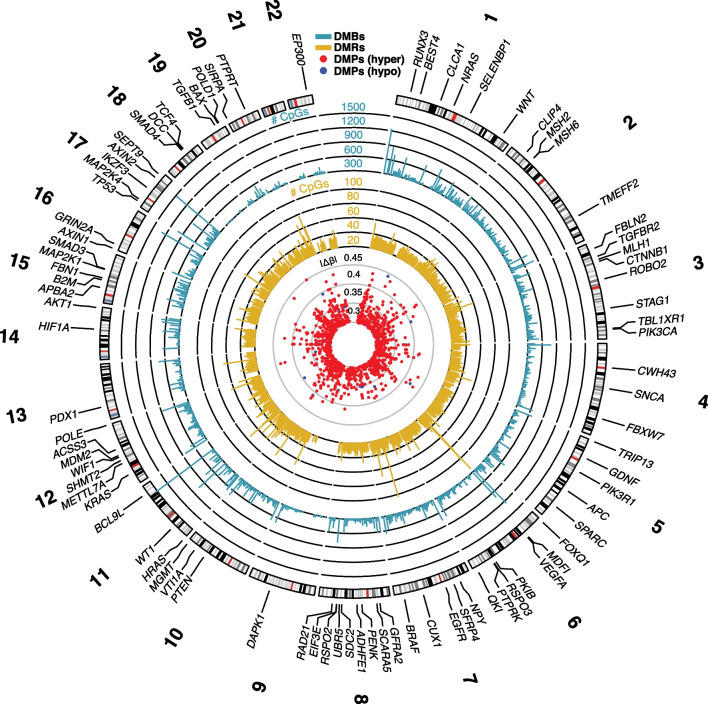
Circular genome plot summarizing genome-wide differential methylation between colorectal adenoma and carcinoma tissue. The outermost track displays DMBs, while the middle track displays DMRs. The innermost track shows DMPs overlapping the displayed DMBs and DMRs, and having a |Δβ|≥ 0.3. CRC-related genes in the COSMIC database and those found in literature, that overlap the mentioned DMBs and DMRs are annotated on the periphery. The height of the bars for DMBs and DMRs represents the number of CpGs in those regions and the vertical position of DMPs represents their |Δβ| in carcinomas. *DMBs* differentially methylated block, *DMRs* differentially methylated regions, *DMPs* differentially methylated probes, *hyper* hypermethylated, *hypo* hypomethylated

To test the discriminatory power of methylation markers in classifying adenomas versus carcinomas, which are the most difficult to resolve clinically, a binary logistic regression model was built using the 13 DE DMPs reported above as predictors. Clustering both the public data (Fig. 4A–C) and the in-house data (Fig. 4D) using the 13 DE DMPs resulted in distinct clusters between adenomas and carcinomas and more unified groupings than using the array data as a whole. Hierarchical clustering revealed that these DMPs were more hypermethylated in adenomas and hypomethylated in carcinomas (Fig. 4A). Clustering the public data could clearly resolve the 2 tissue types, albeit some samples remained doubtful (Fig. 4B, C). Clustering the in-house data fared better, resulting in 2 separate clusters with only 2 of the carcinomas localizing in the adenoma cluster (Fig. 4D). The final model was trained on the public EPIC array data and validated in the in-house experimental methylation datasets (Fig. 1, methods). Importantly, the classifier model reached a cross-validated area under the curve (AUC) of 0.996 and 0.855 in the discovery and validation datasets, respectively. Sensitivities and specificities at different cut-off values for the predicted probabilities are shown through a ROC plot (Fig. 4E). At optimal cut-off, a sensitivity of 96.33% and a specificity of 95.28% for the detection of carcinomas versus adenomas were reached, with an overall accuracy of 95.81% and a misclassification error rate of 4.19%. In the in-house data, the model successfully classified 13 out of 13 adenomas and 7 out of 9 carcinomas. In all, the model exhibited high predictive power and good generalizability across different datasets. The results of the validation of the DE DMPs for comparison of adenoma vs normal and carcinoma vs normal are reported in Additional file 1: Fig. 2 and Fig. 3. In addition, a circos plot representing the genome-wide differential methylation between adenoma and carcinoma tissue is provided in Fig. 5. This plot depicts the DMPs, DMRs and DMBs of A vs C in view of the epigenome and compared to known CRC biomarkers.

### Gene set enrichment analysis

Reactome, gene ontology (GO) and KEGG functional enrichment analysis were performed to better understand the functional implication of differential methylation patterns in adenoma vs carcinoma samples. Pathways were selected based on *p*-values, with a cut-off at < 0.01. We identified 1375, 111 and 32 pathways for GO, Reactome and KEGG analyses in A vs C, respectively (Additional file 3: Table 5). The top 10 most significantly enriched gene sets in each category are represented in Additional file 1: Fig. 4. Functional terms that were highly enriched included terms related to developmental pathways, cell organelles, metabolism, signaling and response mechanisms. Searching for overlapping pathways within the three analyses, the MAPK signaling, cell cycle, ubiquitin-mediated proteolysis, endocytosis and Wnt signaling pathway were found to be significantly enriched. Furthermore, genes within the enriched pathways were investigated in more detail. The *NEU1* gene, which contained 3 DE DMPs for A vs C, was found in all three GSEAs. Pathways including the *NEU1* gene are mentioned in Additional file 1: Table 6. All frequently mutated genes in cancer are registered in the Catalogue of Somatic Mutations in Cancer (COSMIC) database. This list of genes provides valuable insights into the genetic mechanisms underlying cancer. Within the COSMIC genes, 173 genes were found that were present in all three GSEAs (Additional file 4: Table 7). For example, *BRAF*, *HRAS*, *MLH1* and *EGFR* were found to be enriched in the GSEA.

## Discussion and conclusions

Previous research has demonstrated the methylome’s potential for the discovery of biomarkers. In CRC, it has been shown that normal and colorectal cancer tissue, as well as low-grade and high-grade adenomas, can be discriminated based on methylation pattern [5–7]. Therefore, we performed the first study to explore and compare the epigenome of normal colorectal tissue, precancerous lesions (adenomas) and colorectal cancer tissue together and to identify biomarkers that can discriminate between these three tissue types. Based on the current available literature, we hypothesized that each of the three tissue types would be differentially methylated.

Our results are consistent with this hypothesis. We identified numerous DMPs, DMBs and DMRs between the three tissue types (Table 1). The most interesting aspect is that when normal colorectal tissue is compared to adenoma or carcinoma tissue, most of the DMPs were hypomethylated in the tissue type with increasing malignant potential (Fig. 3A, B), which indicates an important role for hypomethylation in carcinogenesis. This is in accordance with previous studies that indicated widespread hypomethylation in cancer tissue compared to healthy tissue, which is observed across cancer types [16, 17]. It also corresponds to the findings of Fan et al*.*, who observed increasing DNA hypomethylation starting from low-grade adenoma stage, leading to further hypomethylation at high-grade adenoma and CRC stage [5]. Likewise, Liu et al. found significantly more hypomethylated DMPs than hypermethylated DMPs in adenoma tissue compared to adjacent normal tissue. For DMRs, the same pattern was observed [18]. When focusing on the difference in methylation between the three tissue types, it is interesting to note that not all normal samples were alike. In the MDS and tSNE plots (Fig. 2C, D), two and four distinct subclusters for the normal samples can be observed, respectively. This indicates the possibility of several subtypes of normal colon tissue with different methylation patterns. We observed different clusters based on sample location (left vs right, data not shown), which has also been described in literature before [19–22]. However, healthy colon tissue adjacent to the tumor tissue was used instead of normal colon samples of healthy patients. In literature, the phenomenon of field cancerization has been described, where amongst others epigenetic changes have been reported in normal colon mucosa adjacent to the tumor [23–27]. Hawthorn et al*.* described chromosomal instability in regions surrounding the tumor as far as 10 cm distal [23]. Park et al*.* described the aberrant methylation of non-adjacent normal-appearing tissue [25]. Unfortunately, for most of the datasets, there is no information on the distance at which the normal-looking tissue was taken, making it difficult to estimate the field effect. However, this clinical information is also lacking in public data. Lastly, two distinct morphological pathways of CRC carcinogenesis exist, potentially explaining the two clusters found in the MDS plot (Fig. 2C). Both the conventional and the alternative/serrated pathways are characterized by specific epigenetic alterations. Different mechanisms lay behind these pathways, which are associated with MSI status and CpG island methylator phenotype (CIMP). A specific CRC classification of five molecular subtypes based on MSI and CIMP status has been described previously. The four distinct clusters found in the tSNE plot (Fig. 2D) could potentially be explained by these molecular subtypes, but this cannot be verified due to the lack of clinical data [28].

When comparing our DMPs to those found in literature, we find many similarities. For example, CpGs in the *ADHFE1* [5]*, SND1*, *OPLAH, TMEM240, NR5A2, TLX2, COL4A1*, *ZFP64* [13], *MYO1G* [29]*, CREB1* [18]*, NPY* and *PENK* [30] genes were also identified in other studies comparing the methylation pattern of healthy colorectal tissue to adenoma and/or carcinoma tissue. Several of these methylation markers can also be appreciated from the circos plot (Fig. 5).

From a clinical perspective, the difference between colorectal adenoma and carcinoma is the most relevant. Therefore, a more in-depth analysis was performed on the difference in methylation between those 2 tissues (Fig. 4). When comparing their methylation patterns, surprisingly 3 out of 13 DE DMPs were located on chromosome six. Chromosome six is a well-known chromosome in oncology. It contains several clinically important proto-oncogenes as well as the major histocompatibility complex. Several genes linked to CRC are located on this chromosome, including *ROS1*, *VEGFA*, *CDKN1A* and *VIP*. A total of 37 797 DMPs was found in the EPIC analysis. 7 810 thereof were in the major histocompatibility complex (MHC). The MHC contains more than 100 genes implicated in the immune response, including HLA-A, HLA-B and HLA-C. These genes and the MHC class I molecules play an important role in the anti-cancer immune response [31]. Downregulation of MHC class I has been observed in 40–90% of cancer types and was often correlated with a worse prognosis [32].

The three DE DMPs on chromosome 6 were located in the TSS1500 (shore) region of the neuraminidase 1 (*NEU1*) gene. This gene encodes a protein that functions as a lysosomal enzyme. It cleaves terminal sialic acid residues from its substrates including glycoproteins/glycolipids. It has no clear cancer-related function, but it is described to play a role in amongst others pathways for the innate immune system, glycosphingolipid metabolism, diseases of glycosylation and synthesis of substrates in N-glycan biosynthesis [33], which were also found to be enriched in the GSEAs (see Additional file 1: Table 6). Furthermore, three publications have already described a link between *NEU1* and CRC. In 2009, Uemura et al*.* reported the regulatory role of *NEU1* in integrin β4-mediated signaling, which led to the suppression of metastasis [34]. Almost a decade later, Forcella et al*.* found that human sialidases are severely dysregulated in several tumors and described their potential application in cancer diagnosis [35]. Jiao et al*.* further underlines the role of *NEU1* in tumorigenesis regulation through several pathways, including immune-mediated tumorigenesis and regulation of vascularization [36]. In addition, two other DE DMPs reported in this study (Fig. 4), are reported in previous methylation studies in CRC. First, *EREG* methylation and subsequent low *EREG* gene expression were correlated with poor response to anti-EGFR therapy in colorectal cancer [19, 37, 38]. Furthermore, *SND1* methylation was identified as one of the top 14 methylation markers for discriminating between CRC and normal tissue in a study by Naumov et al*.* [13].

Later, the 13 DE DMPs detected through the comparison of the methylation pattern of colorectal adenoma and carcinoma were used to build a model that can discriminate between these two lesions (Fig. 4E). These 13 DE DMPs represent the most significant differences between these two tissue types. During the validation of the prediction model in the in-house experimental methylation dataset, an increased error rate was noted (from 4.19% to 11.62%). This might be due to the smaller group of samples in the validation group and/or due to a lower quality of methylation arrays run on FFPE tissue instead of fresh frozen tissue.

When comparing the performance of our model to other methylation models, it is interesting to compare to *SEPT9*. This is the best-known example of DNA methylation as a biomarker in CRC and was commercialized as the EpiProColon® assay. Although the use of this assay has proven effective for CRC detection, it lacks sensitivity for the detection of adenomas. Sensitivities ranging from 11.2% to 31.8% for methylated *SEPT9* in adenomas have been reported [39]. Combinations with other markers, for example *ALX4*, increased the sensitivity to 37%, which shows there is plenty of room for improvement [40]. Our model, combining 13 DE DMPs, yielded a sensitivity of 96% for discriminating adenomas and carcinomas. All 13 adenomas were correctly classified. This is already a major improvement compared to methylated *SEPT9*, although more research and external validation will be needed to prove the superiority of the 13 DE DMPs.

An aspect of working with public data is the lack of quality control. When the data of publicly available methylation array data were analyzed for this study, certain samples included in these datasets were not able to pass quality control and had to be excluded. Therefore, it is advised to download the signal intensity or raw idat files and not β-values, to perform the quality control yourself to ensure adequate quality.

One of the limitations of this study is the use of FFPE material for methylation arrays. A known problem when FFPE samples are used for methylation arrays is the fact that this often results in lower quality data. Previous studies showed that a restore method can result in reliable and high-quality epigenomic data, concordant to that of fresh frozen tissue [41–43]. Therefore, the Infinium HD FFPE Restoration kit was used in this study. However, in our analyses it was noticed that the results of the validation of the 13 DE DMPs were sample dependent. The sample age did not affect the quality of FFPE-derived DNA, which is in concordance with the study of Kling et al*.* [44]. When the model was tested with lower-quality samples (without the expected bimodal distribution of beta values and more beta values around 0.5), it performed worse (data not shown) [44]. High-quality data are thus needed for reliable analyses. Even though different pretreatment processes were developed to reduce formalin artifacts [41], restoring FFPE samples was not found to be effective in our study. This resulted in a limited number of samples used in this study.

Another restriction of this study is that only one out of 13 adenomas from the in-house experimental methylation dataset was high-grade, while all others were low-grade. It would be of interest to identify methylation markers to make a distinction between low-grade and high-grade adenomas, since this might allow for minimally invasive identification of high-grade adenomas, which are known to have a higher risk of developing into carcinoma. However, since only one of our adenoma samples was high-grade and that dysplasia grade was not reported for most adenomas in the public datasets, this analysis was not possible. However, this comparison has been reported by Fan et al*.* [5].

Due to the stability of DNA methylation and the fact that aberrant methylation occurs early in carcinogenesis, the methylome has been considered an ideal source for potential biomarkers. The findings of this study raise the possibility that the 13 DE DMPs identified in this study can be used as targets for a liquid biopsy assay to distinguish adenoma from carcinoma in a minimally invasive way. The non-invasive detection of colorectal adenoma and carcinoma and the distinction between these lesions is highly clinically relevant. Early detection and removal of these lesions in the colorectum can prevent the development and locoregional or metastatic spread of colorectal cancer. Most adenomas and carcinomas are detected through colorectal cancer screening with fecal occult blood tests and subsequent colonoscopy. However, for certain patient groups these tests are not ideal, and a minimally invasive test is preferred. For example, in patients with congestive heart disease the fluid load of bowel preparation should be avoided and in patients who are treated with anticoagulants an invasive colonoscopy with biopsy for histopathological analysis can cause bleeding. Since only a small proportion (± 5%) of adenomas will eventually progress to carcinoma and this process takes up to 5–10 years, the removal of an adenoma is less urgent than the removal of a carcinoma. Therefore, it is of clinical importance to not only detect these lesions minimally invasively, but also to discriminate between these two tissue types, since treatment and follow-up will be different. In addition, a minimally invasive method to do this (e.g., liquid biopsy or stool samples with the 13 DE DMP markers), would be an important added value. With this study, we demonstrate the strength of differentially methylated CpG sites to be used in the clinic as biomarkers. In conclusion, our analyses highlight the power of the methylome, showing that methylation biomarkers can be used to identify colorectal adenoma and carcinoma, but also have the potential to discriminate between these two tissue types.

## Supplementary Information

**Additional file 1:** Supplementary information including Supplemental Tables 1, 2, 3 and 6 and Supplemental Figures 1, 2, 3 and 4.

**Additional file 2:** Supplemental Table 4.

**Additional file 3:** Supplemental Table 5.

**Additional file 4:** Supplemental Table 7.

## Data Availability

The dataset supporting the conclusions of this article is available in the EGA European Genome-Phenome Archive (accession number: EGAS00001007017).
