# Supplementary material for: Epigenome-wide methylation analysis of colorectal carcinoma, adenoma and normal tissue reveals novel biomarkers addressing unmet clinical needs
Source: Clin Epigenetics. 2023 Jul 6;15:111. doi: 10.1186/s13148-023-01516-7 (PMC10327366; doi:10.1186/s13148-023-01516-7)
Supplement: Supplementary file 1 — Additional file 1: Supplementary information including Supplemental Tables 1, 2, 3 and 6 and Supplemental Figures 1, 2, 3 and 4. [file 13148_2023_1516_MOESM1_ESM.docx]

# **Supplementary information**

# 1. Supplementary tables

**Suppl. Table 1. Sample characteristics**

|  | **Normal (19)** | | **Adenoma (17)** | | **Carcinoma (19)** | |
| --- | --- | --- | --- | --- | --- | --- |
| *Characteristic* | *Number* | *%from total* | *Number* | *%from total* | *Number* | *%from total* |
| **Sex** |  |  |  |  |  |  |
| Male | 13 | 68,5 | 9 | 53 | 12 | 63 |
| Female | 6 | 31,5 | 8 | 47 | 7 | 37 |
| **Age** |  |  |  |  |  |  |
| Median | 66,5 | / | 67,5 | / | 75,5 | / |
| Interval | 50 - 83 | / | 50 - 82 | / | 56-95 | / |
| **Location** |  |  |  |  |  |  |
| Left | 11 | 57,9 | 10 | 59 | 11 | 57,9 |
| Right | 5 | 26,3 | 6 | 35 | 5 | 26,3 |
| Unknown | 3 | 15,8 | 1 | 6 | 3 | 15,8 |
| **Dysplasia** |  |  |  |  |  |  |
| Low-grade | / | / | 15 | 88 | / | / |
| High-grade | / | / | 2 | 12 | / | / |
| **Type** |  |  |  |  |  |  |
| Tubulo-villous | / | / | 5 | 29 | / | / |
| Tubular | / | / | 12 | 71 | / | / |

* This included paired samples from the same patient: 10 tumor-normal pairs and 1 adenoma-normal pair

**Suppl. Table 2. Samples that passed quality control of the in-house generated methylation data**

| **Tissue type** | **Concentration (ng/µL)** | **Gender** | **Age** |
| --- | --- | --- | --- |
| Nl | 60,8 | Male | 83 |
| LGA (Tb) | 33,6 | Female | 70 |
| CRC | 187 | Female | 72 |
| CRC | 155 | Male | 71 |
| HGA (Tv) | 31,7 | Male | 64 |
| Nl | 22,7 | Male | 63 |
| CRC | 445 | Male | 76 |
| LGA (Tv) | 21,5 | Female | 50 |
| LGA (Tv) | 41,5 | Male | 70 |
| CRC ^1^ | 148 | Female | 62 |
| LGA (Tb) | 41,4 | Female | 82 |
| LGA (Tb) | 17,4 | Female | 68 |
| CRC | 118 | Female | 71 |
| LGA (Tb) | 23,2 | Male | 73 |
| LGA (Tv) | 114 | Male | 66 |
| LGA (Tb) | 31,8 | Male | 64 |
| Nl ^1^ | 63,2 | Female | 62 |
| LGA (Tb) | 20 | Male | 73 |
| LGA (Tb) | 23,4 | Male | 66 |
| LGA (Tb) | 46,3 | Female | 77 |
| CRC | 56,4 | Female | 72 |
| LGA (Tb) | 29,8 | Male | 67 |
| CRC | 29,1 | Male | 76 |
| CRC ^2^ | 298 | Male | 59 |
| CRC | 155 | Female | 68 |
| Nl ^2^ | 46,2 | Male | 59 |
| Nl | 61,3 | Male | 79 |

(Nl = normal adjacent, CRC= colorectal cancer, LGA = low grade adenoma, HGA = high grade adenoma, Tb = tubular, Tv= tubulovillous, ^1,2...^ = paired sample)

**Suppl. Table 4: Location of DE DMPs in different groups within the genome**

Due to the large number of rows, the table is uploaded in a separate file to facilitate access for readers.

**Suppl. Table 3: Overview of the datasets used for discovery, double evidencing and validation.**

| **Dataset ID** | **Adenoma** | **Carcinoma** | **Normal** | **QC Passed Sum** | **Cohort** |
| --- | --- | --- | --- | --- | --- |
| **EMD_01** | 3 | 2 | 3 | 4 | Validation |
| **EMD_02** | 3 | 2 | 3 | 3 | Validation |
| **EMD_03** | 3 | 3 | 2 | 4 | Validation |
| **EMD_04** | 3 | 3 | 2 | 4 | Validation |
| **EMD_05** | 4 | 2 | 2 | 4 | Validation |
| **EMD_06** | 1 | 1 | 1 | 2 | Validation |
| **EMD_07** | 0 | 4 | 4 | 6 | Validation |
| **EMD_08** | 0 | 2 | 2 | 0 | Validation |
| **E-MTAB-6450** | 16 | 0 | 0 | 16 | Discovery/DE |
| **E-MTAB-7854** | 80 | 0 | 0 | 78 | Discovery/DE |
| **GSE132804** | 0 | 0 | 206 | 206 | Discovery/DE |
| **GSE151732** | 0 | 0 | 256 | 256 | Discovery/DE |
| **GSE166212** | 10 | 32 | 6 | 47 | Discovery/DE |
| **GSE199057** | 0 | 77 | 80 | 156 | Discovery/DE |
| **Sum** | **123** | **128** | **567** | **786** |  |
| **GSE106556** | 0 | 0 | 10 | 10 | DE |
| **GSE129364** | 69 | 0 | 3 | 72 | DE |
| **GSE132804** | 0 | 0 | 128 | 128 | DE |
| **GSE139404** | 40 | 0 | 20 | 60 | DE |
| **GSE66555** | 0 | 0 | 43 | 34 | DE |
| **GSE68060** | 0 | 82 | 36 | 118 | DE |
| **GSE77718** | 0 | 96 | 96 | 191 | DE |
| **GSE77955** | 12 | 13 | 14 | 39 | DE |
| **TCGA_READ + COAD)** | 0 | 409 | 45 | 454 | DE |
| **Sum** | **121** | **600** | **395** | **1106** |  |

*Legend: EMD = experimental methylation data, E-MTAB= data from array express, GSE= data from GEO. Green = EPIC array data, blue = 450K array data.*

**Suppl. Table 5: List of** **significantly enriched gene sets based on functional enrichment analysis of DMPs in adenoma versus carcinoma samples.**

Due to the large number of rows, the table is uploaded in a separate file to facilitate access for readers.

**Suppl. Table 6: Pathways in GSEA that contain the *NEU1* gene**

| **Description** | **GSEA** | **ID** |
| --- | --- | --- |
| Lysosome | KEGG | 4142 |
| neutrophil activation involved in immune response | GO | GO:0002283 |
| neutrophil mediated immunity | GO | GO:0002446 |
| lysosomal membrane | GO | GO:0005765 |
| vacuolar membrane | GO | GO:0005774 |
| carbohydrate catabolic process | GO | GO:0016052 |
| granulocyte activation | GO | GO:0036230 |
| neutrophil activation | GO | GO:0042119 |
| neutrophil degranulation | GO | GO:0043312 |
| lytic vacuole membrane | GO | GO:0098852 |
| carbohydrate derivative catabolic process | GO | GO:1901136 |
| vacuolar lumen | GO | GO:0005775 |
| specific granule | GO | GO:0042581 |
| sphingolipid metabolic process | GO | GO:0006665 |
| secretory granule lumen | GO | GO:0034774 |
| Diseases of glycosylation | Reactome | R-HSA-3781865 |
| Asparagine N-linked glycosylation | Reactome | R-HSA-446203 |
| Neutrophil degranulation | Reactome | R-HSA-6798695 |

**Suppl. Table 7:** **Overlap between COSMIC genes and significantly enriched genes from KEGG, GO & Reactome gene sets for adenoma versus carcinoma.**

Due to the large number of rows, the table is uploaded in a separate file to facilitate access for readers.

# **2. Supplemental figures**


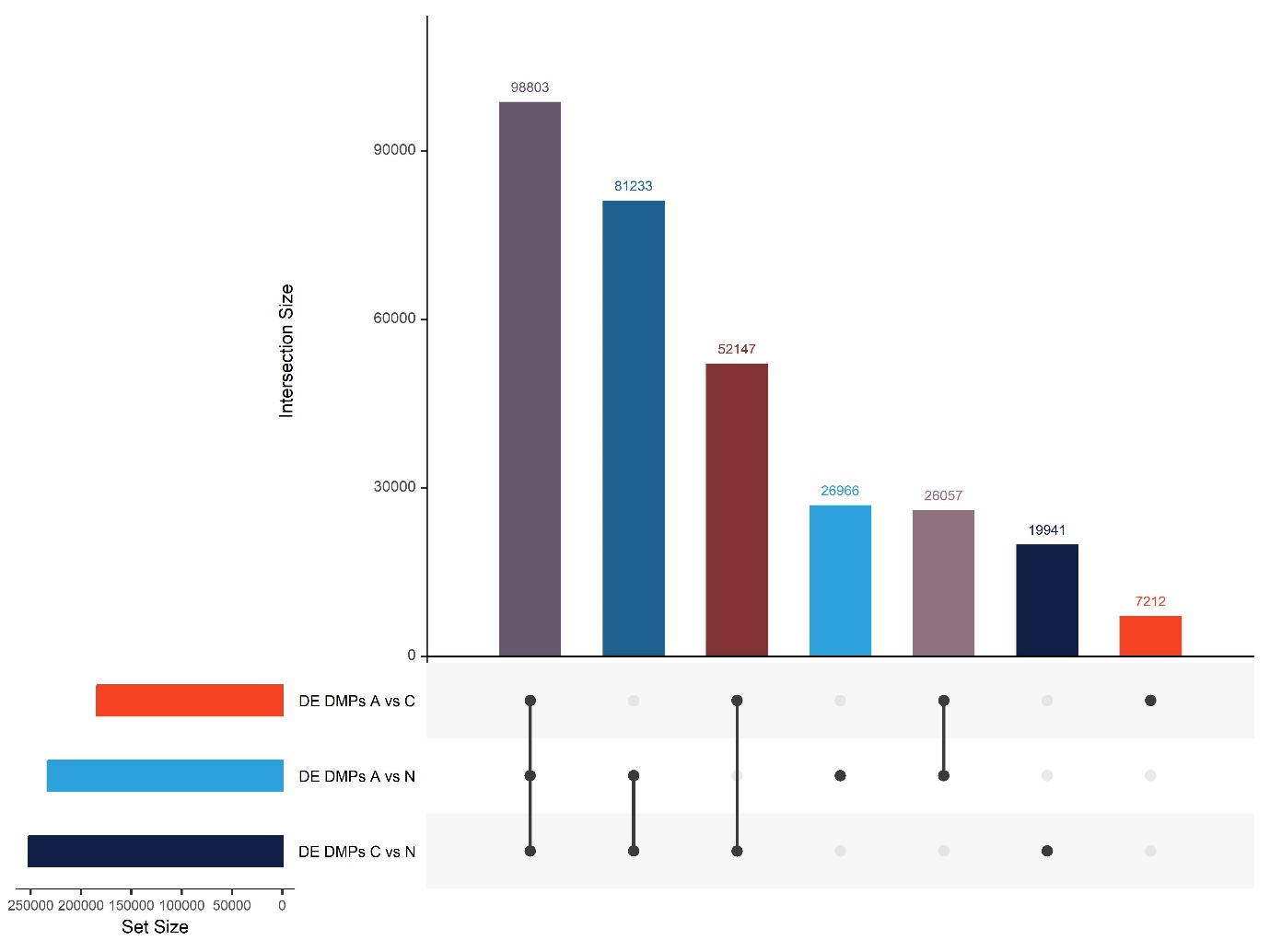


**Suppl. Figure 1. Counts of unique and common DE-DMPs in the three different colorectal tissue types.** The upset diagram shows the total number of DE DMPs found in the discovery analysis prior to filtering based on |Δβ|. Legend: A= adenoma, N= normal, C= carcinoma

#
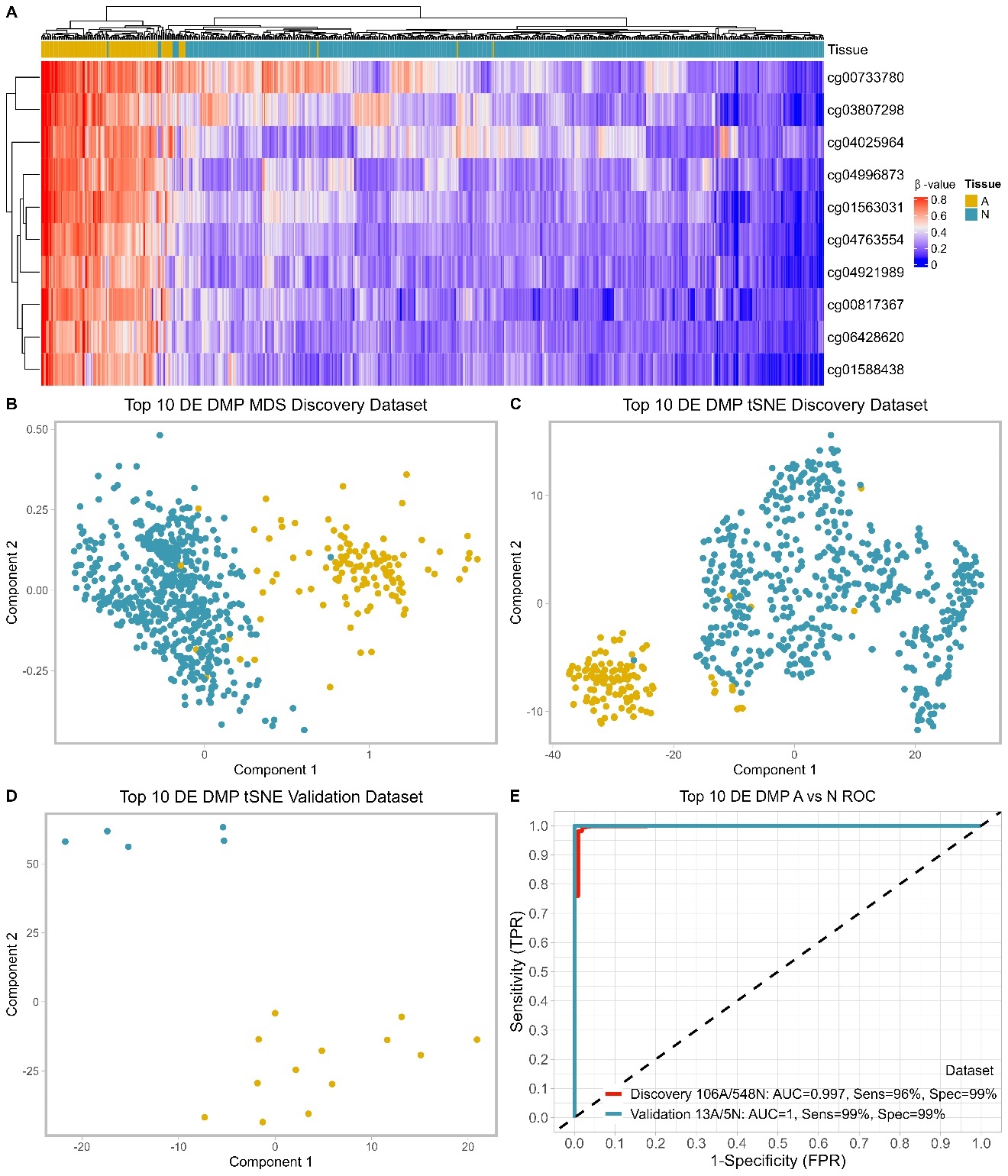


**Suppl. Figure 2. The top 10 selected DE DMP markers were effective at classifying adenomas and normal tissues. A.** Heatmap and hierarchal clustering analysis of the discovery EPIC dataset based on the top 10 identified DE DMP markers shows a block like structure with most of the markers being hypermethylated in adenomas and hypomethylated in normal tissues. **B.** MDS clustering of the discovery dataset using the 10 markers shows 2 distinct clusters. **C.** tSNE clustering of the discovery dataset using the 10 markers could also resolve the two tissue types. **D.** tSNE clustering of the validation dataset using the 10 markers shows a clear separation between adenomas and normal tissues. **E.** ROC curves for the final top 10 DE DMP classifier model for both discovery and validation datasets from EPIC arrays. Sensitivity and specificity, for distinguishing between adenomas and normal tissues, at various cut-off values for the datasets are plotted. The diagonal dotted line represents the line of no discrimination between tumor and normal tissues. DE DMP: double evidenced differentially methylated probes, ROC: receiver operating characteristic, MDS: multidimensional scaling, tSNE: t-distributed stochastic neighbor embedding, TPR: true positive rate, FPR: false positive rate, A: adenoma, N: normal tissue.

**
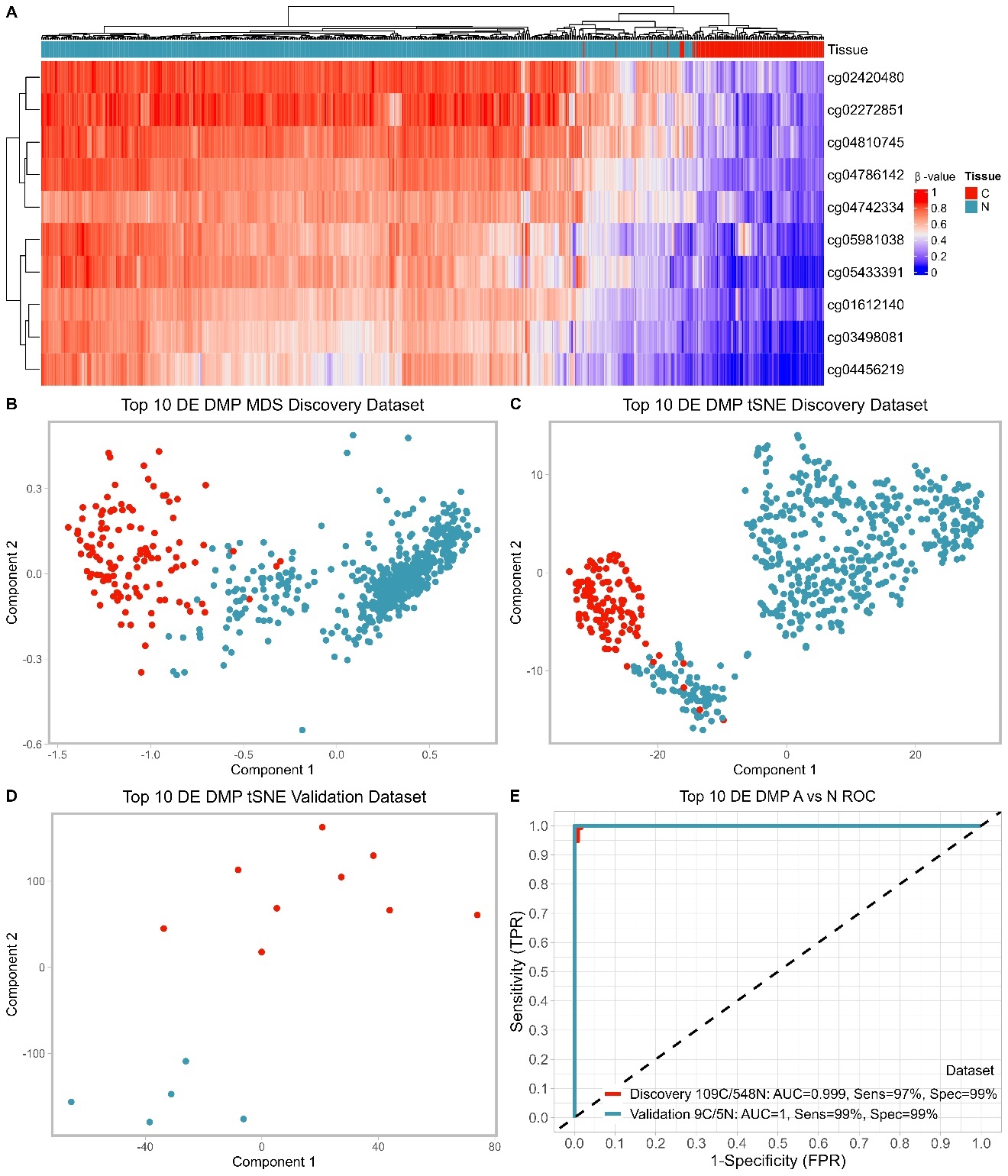
**

**Suppl. Figure 3. The top 10 selected DE DMP markers were effective at classifying carcinomas and normal tissues. A.** Heatmap and hierarchal clustering analysis of the discovery EPIC dataset based on the top 10 identified DE DMP markers shows a block like structure with most of the markers being hypomethylated in carcinomas and hypermethylated in normal tissues. **B.** MDS clustering of the discovery dataset using the 10 markers shows 2 distinct clusters. **C.** tSNE clustering of the discovery dataset using the 10 markers could also resolve the two tissue types. **D.** tSNE clustering of the validation dataset using the 10 markers shows a clear separation between carcinomas and normal tissues. **E.** ROC curves for the final top 10 DE DMP classifier model for both discovery and validation datasets from EPIC arrays. Sensitivity and specificity, for distinguishing between carcinomas and normal tissues, at various cut-off values for the datasets are plotted. The diagonal dotted line represents the line of no discrimination between tumor and normal tissues. DE DMP: double evidenced differentially methylated probes, ROC: receiver operating characteristic, MDS: multidimensional scaling, tSNE: t-distributed stochastic neighbor embedding, TPR: true positive rate, FPR: false positive rate, C: carcinoma, N: normal tissue.

**
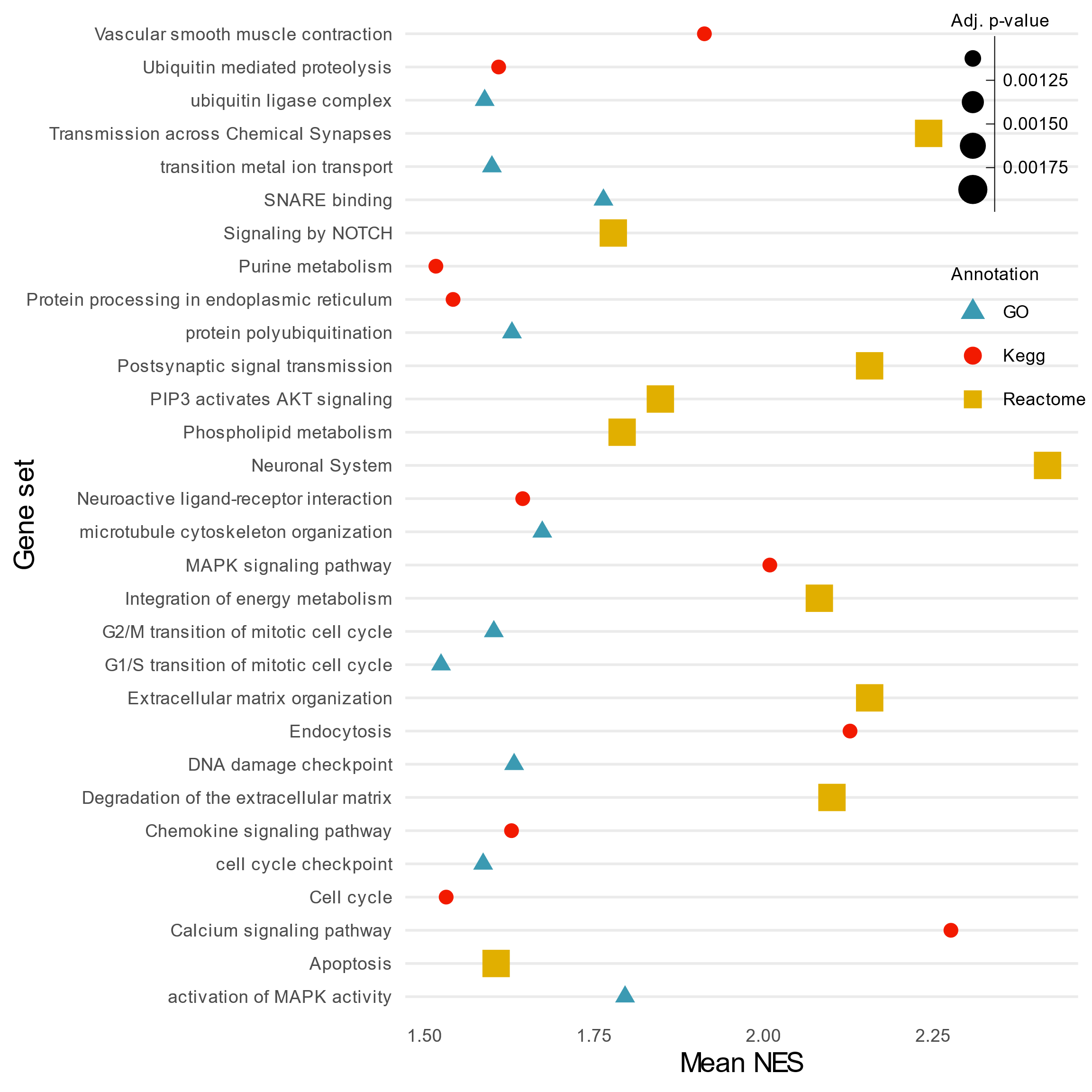
**

**Suppl. Figure 4. Dot plot outlining the gene set enrichment analysis (GSEA) results based on the GO, KEGG, and Reactome gene sets.** The top 10 most significantly enriched gene sets (FDR ≤ 0.05) in each category are shown. The dot size corresponds to the adjusted p-value of each gene set while the normalized enrichment score (NES) is shown on the x-axis.
